# Loss of Hormone Receptor Expression after Exposure to Fluid Shear Stress in Breast Cancer Cell Lines

**DOI:** 10.3390/ijms25137119

**Published:** 2024-06-28

**Authors:** Jonathan Cuccia, Braulio Andrés Ortega Quesada, Ethan P. Littlefield, Alejandra M. Ham, Matthew E. Burow, Adam T. Melvin, Elizabeth C. Martin

**Affiliations:** 1 Biological and Agricultural Engineering, Louisiana State University, Baton Rouge, LA 70803, USA; jcucci@lsuhsc.edu (J.C.); elittl5@lsu.edu (E.P.L.); aham4@lsu.edu (A.M.H.); 2 Department of Chemical and Biological Engineering, Clemson University, Clemson, SC 29634, USA; braulio@clemson.edu (B.A.O.Q.); melvina@clemson.edu (A.T.M.); 3 Department of Medicine, Section Hematology and Medical Oncology, Tulane University, New Orleans, LA 70118, USA; mburow@tulane.edu; 4 Tulane University Cancer Center, Tulane University, New Orleans, LA 70118, USA

**Keywords:** metastatic breast cancer, hormone receptor positive breast cancer, fluid shear stress, proteomics

## Abstract

Following metastatic spread, many hormone receptor positive (HR^+^) patients develop a more aggressive phenotype with an observed loss of the HRs estrogen receptor (ER) and progesterone receptor (PR). During metastasis, breast cancer cells are exposed to high magnitudes of fluid shear stress (FSS). Unfortunately, the role for FSS on the regulation of HR expression and function during metastasis is not fully understood. This study was designed to elucidate the impact of FSS on HR^+^ breast cancer. Utilizing a microfluidic platform capable of exposing breast cancer cells to FSS that mimics in situ conditions, we demonstrate the impact of FSS exposure on representative HR^+^ breast cancer cell lines through protein and gene expression analysis. Proteomics results demonstrated that 540 total proteins and 1473 phospho-proteins significantly changed due to FSS exposure and pathways of interest included early and late estrogen response. The impact of FSS on response to 17β-estradiol (E2) was next evaluated and gene expression analysis revealed repression of ER and E2-mediated genes (*PR* and *SDF1*) following exposure to FSS. Western blot demonstrated enhanced phosphorylation of mTOR following exposure to FSS. Taken together, these studies provide initial insight into the effects of FSS on HR signaling in metastatic breast cancer.

## 1. Introduction

Metastatic cancer is associated with poor patient prognoses and drug resistance [1]. This is especially critical in the histologically identified hormone receptor positive (HR^+^) breast cancer subtype, which accounts for approximately ~70% of breast cancer cases and relies on the estrogen receptor alpha (ER) for proliferation and tumor growth [2]. Although primary HR^+^ tumors are known to be responsive to endocrine therapy, once metastasized, these tumors are more aggressive and less sensitive to standard of care endocrine therapies. The more aggressive behavior observed in HR^+^ metastatic breast cancer is hypothesized to occur through multiple mechanisms including: (***i***) loss of the estrogen receptor (ER), (***ii***) acquisition of additional mutations, and/or (***iii***) alterations in estrogen and growth factor-mediated signaling cascades [3,4]. There is a need to better understand the mechanisms driving HR^+^ breast cancer growth and survival following metastasis as metastatic breast cancer accounts for 90% of cancer-related deaths [1]. Development of macro-metastasis is an inefficient process, with only a minority of breast cancer cells successfully establishing at distal tissue sites [5]. Growth at secondary sites requires the acquisition of abilities that promote survival in a new unfavorable microenvironment. Current studies suggest the regulation of growth and survival pathways as well as cytokine release [5]. To date, it is unclear if these pro-survival adaptations were present in the primary tumor or acquired during metastatic spread in the vasculature. Current in vitro methods to study cancer metastasis are designed to interrogate cells during the initial stages of the cascade (e.g., invasion of basement membrane) [6]. There are limited approaches to study the later stages of the metastatic cascade in vitro. Specifically, there is a lack of pre-clinical models that allow for the interrogation of biophysical forces exerted on circulating cancer cells, such as fluid shear stress (FSS), the unit area of force acting on a cell in the vasculature, which can cause genotypic and phenotypic alterations in cancer. Physiological shear stress magnitudes vary greatly depending on the location of the measurement from almost 0 dyn/cm^2^ in the microcirculation to ~120 dyn/cm^2^ in the great arteries of the heart. The average FSS magnitudes imposed upon circulating cells are 1–6 dyn/cm^2^ and 15–20 dyn/cm^2^ for venous and arterial flow, respectively. Most studies investigating how exposure to FSS changes cellular phenotypes involve flowing cells through micro-tubing using either a syringe pump or a peristaltic pump [7,8,9,10]. While effective, the large diameter of tubing (~500 µm–10 mm) limits the ability to deliver uniform magnitudes of FSS to individual cells. Microfluidic devices are a superior alternative to overcome this limitation and provide a greater degree of control on the durations and magnitudes of applied FSS to single cells. Prior studies examined how FSS exposure affects endothelial cell elongation and proliferation [11,12], tumor-endothelial cell interaction [13], cellular deformation [14,15], drug toxicity [16], FSS-mediated epithelial to mesenchymal transition (EMT) and cancer stem cell (CSC) biology [17,18,19,20]. Prior studies on breast cancer have utilized the triple negative breast cancer cell line MDA-MB-231 or the hormone receptor positive cell line MCF-7. Collectively, the influence of FSS on HR^+^ breast cancer is understudied. There is a lack of studies designed to determine the influence of FSS on HR^+^ breast cancer both immediately after exposure to FSS and following extended time points in culture. End point analysis of prior studies focused on FSS were primarily obtained directly after exposure to FSS, with one study prolonging growth to 1 week [17,18,19,21]. We previously employed a microfluidic approach to examine FSS-induced deformation of breast cancer and confirmed significant heterogeneity in the single cell response [15]. This study focused on biophysical changes in cells due to exposure to FSS and neglected both the role of FSS on intracellular signaling and the long-term impact. To address this limitation, we recently designed and utilized a modular microfluidic device that effectively and accurately recapitulates the fluid shear stress that metastasizing breast cancer cells experience while circulating through the human vasculature [22]. The modular system consists of two separate microfluidic devices, including a shearing device containing a single fluidic channel capable of delivering uniform magnitudes of FSS to cells that mimic the human vasculature and a microwell trapping array capable of isolating and studying single cancer cells. A unique feature of this device was the ability to perform both on-chip single cell immunostaining (using the two devices) or off-chip bulk analysis with PCR or Western blotting (using only the shearing device). The study performed here expands on our initial work to better define how FSS alters intracellular signaling in HR^+^ breast cancer.

## 2. Results

### 2.1. Exposure to Fluid Shear Stress Enhances Phospho-Proteins Associated with Cell Death and DNA Damage Response in HR^+^ Breast Cancer Cells

**Figure 1:**
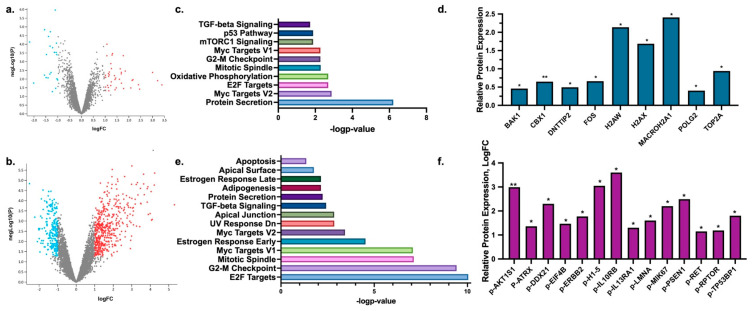
Fluid shear stress activates cell death and DNA damage response in hormone receptor positive breast cancer cells. Volcano plot of significantly altered total- (**a**) and phospho- (**b**) proteins changed in MCF-7 cells following exposure to FSS. Significantly altered pathways as identified by Enrichr Hallmark pathways for total (**c**) and phospho- (**e**) proteins changed. Select total (**d**) and phospho- (**f**) proteins of interest observed to be upregulated following exposure to FSS. Comparison was to non-FSS-exposed MCF-7 cells maintained in suspension. N = 5 biological replicates * *p* < 0.05 and ** *p* < 0.01.

We have previously utilized the modular microfluidic platform to perform single cell analysis on FSS-induced changes in markers of proliferation or protein phosphorylation [22]. We demonstrated that exposure to FSS elevated phospho-proteins in the AKT/mTOR signaling pathways immediately after exposure to FSS [22]. To better understand the full scope of intracellular signaling cascades activated by FSS, we expanded on this work and exposed the HR^+^ breast cancer cell line, MCF-7, to 10 dyn/cm^2^ FSS using only the shearing device and performed total and phospho-proteomics immediately after exposure to FSS. Comparisons were made to non-shear control MCF-7 cells that were maintained in suspension but not exposed to FSS. Results demonstrated 540 total proteins and 1473 phospho-proteins significantly changed following exposure to FSS (Figure 1a,b). Pathway analysis was performed in Enrichr [23,24,25] to determine trends in pathways altered by FSS exposure for both phospho- and total proteins changed. Shared pathways of interest for both phospho- and total proteins included mitotic spindle, G2-M checkpoint, and TGFβ signaling (Figure 1c,d). Unique pathways of interest for total protein changes demonstrated alterations to the p53 pathway, mTORC1 signaling, and oxidative phosphorylation. Overall, there was a trend for total protein changes to be associated with pathways commonly altered with cell death and DNA-damage respone. An in-depth evaluation of total proteins enhanced following exposure to FSS demonstrated increased total protein expression linked to these processes, such as BAK1, CBX1, DNTTIP2, FOS, H2AW, H2AX, MACROH2A1, POLG2, and TOP2A (Figure 1d). Significantly altered phospho-proteins demonstrated a similar trend favoring pathways commonly altered with cell death and DNA-damage response with observed changes in proteins association with the UV response and apoptosis pathways (Figure 1e). Similarly to total protein changes, phosphorylation of proteins that directly regulated DNA (p-ATRX, p-DDX21, p-H1–5, p-TP53BP1) was also observed; however, the phospho-proteomics also demonstrated enrichment of proteins commonly associated with growth factor and extracellular signaling responses (p-AKT1S1, p-ATP2B1, p-EIF4B, p-ERBB2, p-IL10RB, p-IL13RA1, p-LMNA, p-MIK67, p-PSEN1, p-RET, p-RPTOR) (Figure 1f). Notably, there was an enrichment in phospho-proteins associated with the mTOR signaling cascade and an increase in phospho-proteins associated with early and late estrogen responses, which was not observed in the total protein changes (Figure 1e).

### 2.2. Exposure to Fluid Shear Stress Regulates HR Expression in MCF-7 Breast Cancer Cells

**Figure 2:**
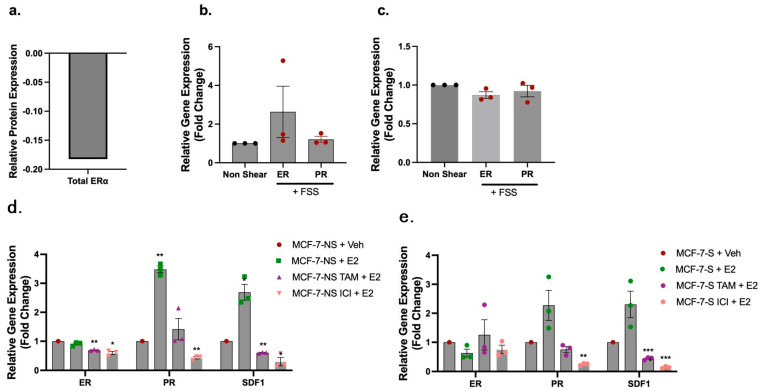
Exposure to fluid shear stress alters the transcriptional response to endocrine treatment in hormone receptor positive breast cancer. (**a**) Protein expression of ER immediately after exposure to FSS. (**b**,**c**) Gene expression of *ER* and *PR* immediately after (**b**) or 24 h after (**c**) exposure to 10 dyn/cm^2^ FSS. (**d**,**e**) Gene expression for *ER*, *PR*, and *SDF1* in MCF-7 cells exposed or not exposed to FSS followed by 24 h treatment with vehicle control (DMSO), 17-βestradiol (E2), or pre-treatment with tamoxifen or fulvestrant (ICI) prior to stimulation with E2. Error bars represent SEM and * *p* < 0.05, ** *p* < 0.01, and *** *p* < 0.001. NS = non-FSS-exposed MCF-7 cells maintained in suspension but not exposed to FSS. S = FSS-exposed MCF-7 cells.

Proteomics data revealed alterations to proteins associated with late and early estrogen response; however, there were no observed changes to total ER protein levels immediately after exposure to FSS (Figure 2a) and phospho-ER was not detected at any known phospho-site. To gain an increased understanding of the effects of FSS exposure on HR expression and function, MCF-7 cells were exposed to FSS as described above and then either collected immediately for gene expression analysis or seeded on tissue culture plastic (TCP) and maintained in culture. Non-shear control MCF-7 cells were maintained in suspension but not exposed to FSS; similarly to FSS-exposed cells, cells were collected immediately or seeded on TCP and maintained in culture. Shear and non-shear-exposed cells were collected at 24 h post exposure to FSS. Gene expression analysis was performed for *ER* and the ER-mediated gene *PR*. Results demonstrated no change in gene expression immediately after exposure to FSS or at 24 h post exposure to FSS compared to non-FSS-exposed control cells (Figure 2b,c). To determine if FSS altered estrogen-mediated ER-regulated genes in HR positive cells, we next exposed MCF-7 cells to FSS and then seeded the cells in 5% dextran stripped FBS media. After 24 h of culture, cells were treated with 17β-estradiol (E2) for 24 h to determine alterations in E2-mediated gene expression. After E2 treatment, control non-shear-exposed MCF-7 cells demonstrated the expected significant increase in expression of ER-mediated genes *PR* and *SDF1* (Figure 2d), with increases in expression of factors of 3.48 ± 0.11 and 2.69 ± 0.27, respectively. In contrast, MCF-7 cells exposed to FSS did not demonstrate a significant change in gene expression for either PR or *SDF1* (Figure 2e). Further, while pre-treatment with tamoxifen and ICI significantly repressed *ER* expression in the non-shear-exposed MCF-7 cells by 0.69 ± 0.01 and 0.59 ± 0.06, respectively, there was no change in *ER* expression with endocrine treatment in FSS-exposed cells (Figure 2d,e). Both non-shear and FSS-exposed MCF-7 cells demonstrated significant repression of ER-mediated gene expression for *PR* and *SDF1* with treatment of endocrine inhibitors (Figure 2d,e). We next sought to determine if FSS altered proliferation of HR^+^ breast cancer cells. MCF-7 cells were grown in media free of exogenous estrogens and, after 24 h, cells were exposed to FSS and then immediately plated in a 96 well plate. Cells were given 24 h to adhere and then treated with E2, tamoxifen, or ICI for 3 days. Despite the observed changes in ER-mediated genes post exposure to FSS, MCF-7 cells demonstrated no change in basal proliferation with endocrine treatment (Supplementary Figure S1).

**Figure 3:**
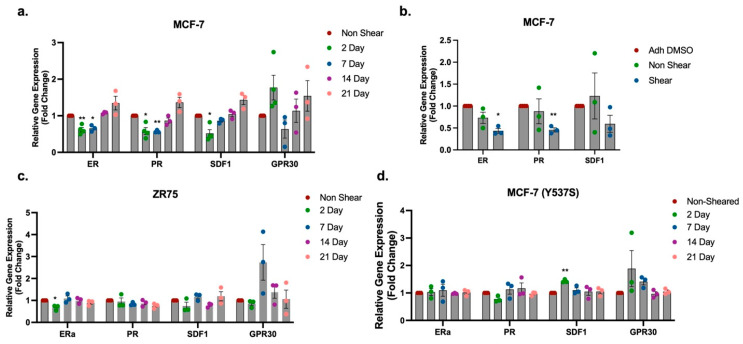
Exposure to fluid shear stress represses estrogen receptor expression in hormone receptor positive breast cancer. (**a**–**d**) Gene expression for *ER*, *PR*, *SDF1*, and *GPR30* in HR positive breast cancer cell lines MCF-7 (**a**,**b**), ZR-75 (**c**), and (**d**) MCF-7-Y537S after exposure to FSS followed by growth in culture on TCP for 2, 7, 14 and 21 days. Normalization was to non-sheared MCF-7 cells in suspension (**a**,**c**,**d**) or non-sheared and adherent MCF-7 cells (**b**). Error bars represent SEM and * *p* < 0.05 and ** *p* < 0.01.

Due to the observed alterations in endocrine-mediated HR expression after exposure to FSS, the long-term impact of FSS on HR expression in MCF-7 cells was evaluated next. To achieve this, MCF-7 cells were exposed to 10 dyn/cm^2^ of FSS and then seeded in culture on TCP in normal growth media. Non-shear control MCF-7 cells were maintained in suspension but not exposed to FSS; similarly to FSS-exposed cells, non-shear MCF-7 cells were seeded on TCP and maintained in culture. Following a period of growth, shear-exposed and non-shear-exposed cells were collected at intervals of 48 h and 1, 2, and 3 weeks and analyzed for gene expression changes. At 48 h, gene expression for *ER* (0.62 ± 0.05), *PR* (0.58 ± 0.09), and *SDF1* (0.52 ± 0.10) was significantly down-regulated in MCF-7 cells exposed to FSS compared to non-sheared control cells (Figure 3a). Repression of *ER* (0.65 ± 0.05) and *PR* (0.57 ± 0.02), but not *SDF1* (0.86 ± 0.37) was sustained at 1 week post exposure to FSS (Figure 3a). When evaluated at 2 and 3 weeks post exposure to FSS, *ER*, *PR*, and *SDF1* demonstrated no significant change in gene expression compared to non-sheared MCF-7 control cells (Figure 3a). Analysis of the non-genomic ER receptor, *GPR30*, demonstrated no significant change in gene expression after exposure to FSS at 48 h or longer periods in culture, suggesting that the impact of FSS exposure may be specific to ER. To further demonstrate that the observed repression in HR gene expression is mediated by the forces exerted by FSS, and not due to loss of adhesion while in suspension, *ER* and *PR* gene expression for non-shear suspended and shear-exposed MCF-7 cells was compared to that of MCF-7 cells maintained on TCP without a period of suspension. Results demonstrated that non-shear-exposed MCF-7 had gene expression levels like adherent MCF-7 cells, while FSS-exposed cells had a loss of *ER* and *PR* with changes in gene expression of factors of 0.44 ± 0.05 and 0.46 ± 0.05, respectively. We next evaluated HR and *SDF1* gene expression in two additional cell lines, ZR-75 and MCF-7-Y537S, an MCF-7 variant with CRISPR-Cas9 genome editing to insert a constitutively active ER mutation [26]. Results from the ZR-75 cell line demonstrated a significant repression of *ER* (0.68 ± 0.06) at 48 h. HR expression and *SDF1* expression were not significantly different at any other time point (Figure 3c). The MCF-7-Y537S cell line demonstrated no change in HR expression at any time point. *SDF1* gene expression was significantly enhanced at 2 days post exposure to FSS (Figure 3d). Taken together, this suggests that exposure to FSS mediates ER expression in cell lines with wild type (WT) ER but not in ER mutant lines.

### 2.3. Exposure to Fluid Shear Stress Induces Activation of mTOR Signaling in HR^+^ Breast Cancer Cells

**Figure 4:**
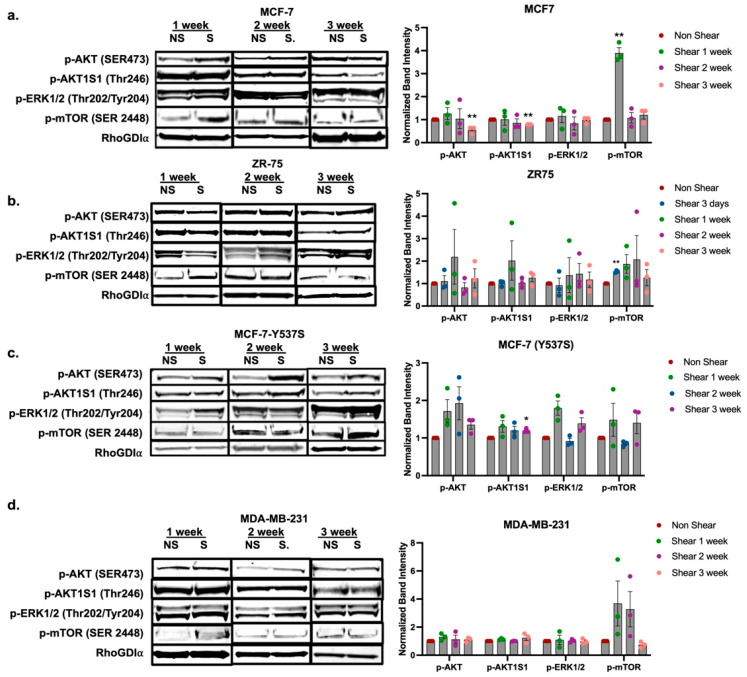
Exposure to fluid shear stress induces activation of mTOR signaling in hormone receptor positive breast cancer cells. Western blot analysis for MCF-7 (**a**), ZR-75 (**b**), MCF-7-Y537S (**c**), and MDA-MB-231 (**d**) cell lines for p-AKT, p-AKT1S1, p-ERk1/2, and p-mTOR after exposure to FSS followed by growth in culture on TCP for 1, 2, and 3 weeks. While in culture, cells were not exposed to FSS. Normalization was to loading control (RhoGDI) and non-FSS-exposed MCF-7 control cells. Non-FSS controls cells were maintained in suspension but not exposed to FSS prior to seeding on TCP. Error bars represent SEM and * *p* < 0.05 and ** *p* < 0.01.

Our phospho-proteomics data demonstrated increased phosphorylation of growth factor-mediated extracellular receptors such as p-RET and p-ERBB2 and downstream signaling kinases p-AKT1S1, p-RPTOR, and p-EIF4B (Figure 1f), proteins which have a known association with altered ER signaling through AKT/mTOR crosstalk [27,28]. Previous work using the modular microfluidic device demonstrated elevated p-AKT (Ser473) and p-mTOR (Ser2448), but not p-ER (Ser167), directly after exposure to FSS [22]. Due to this, and the observed repression of HR expression following exposure to FSS, we next sought to determine if FSS induced long-term activation of the AKT/mTOR signaling axis. Western blot analysis was performed for p-AKT (Ser473), p-AKT1S1 (Thr246), and p-mTOR (Ser2448) in the HR^+^ breast cancer cell lines MCF-7, ZR-75, and MCF-7-Y537S following exposure to 10 dyn/cm^2^ of FSS using the microfluidic device followed by seeding in culture and growth on TCP. Non-shear control MCF-7 cells were maintained in suspension but not exposed to FSS; similarly to FSS-exposed cells, cells were seeded on TCP and maintained in culture. Following a period of growth, shear-exposed and non-shear-exposed cells were collected at intervals of 1, 2, and 3 weeks. MAPK activation was also evaluated by expression of p-ERK1/2 (Thr202/Tyr204), as it was previously demonstrated to be repressed by exposure to FSS [22]. Following 1 week on TCP post-exposure to FSS, p-mTOR (Ser2448) was significantly elevated by a factor of 3.89 ± 0.23 in the MCF-7 cell line compared to the non-sheared control cells (Figure 4a). p-mTOR levels were normalized to that of the non-sheared MCF-7 cells at 2 weeks post exposure to FSS. There was no observed increase in either p-AKT (Ser473) or p-AKT1S1 (Thr246) in MCF-7 cells exposed to FSS after 1 week in culture; furthermore, p-AKT (Ser473) and p-AKT1S1 (Thr246) were significantly repressed by 0.56 ± 0.09 and 0.79 ± 0.001, respectively, at 3 weeks compared to non-sheared control cells (Figure 4a). There was no observed change in p-ERK1/2 (Thr202/Tyr204) at any time point in MCF-7 cells exposed to FSS. The ZR-75 cell line demonstrated no change in p-AKT (Ser473), p-AKT1S1 (Thr246), p-mTOR (Ser2448), or p-ERK1/2 (Thr202/Tyr204) following exposure to FSS and growth on TCP for 1, 2, and 3 weeks. p-mTOR (Ser2448) demonstrated elevated phosphorylation at 1 week; however, the factor increase was variable for each replicate and started to decrease at 2 and 3 weeks post exposure to FSS. The MCF-7-Y537S cell line demonstrated no change in phospho-proteins at any time point (Figure 4c). p-AKT (Ser473) and p-ERK1/2 (Thr202/Tyr204) were elevated at 1 week; however, the magnitude of enhanced expression was variable and therefore not significant. This further suggests that the effects of FSS exposure on HR^+^ cells may be specific to WT ER cell lines. To determine if activation of p-mTOR occurred in breast cancer subtypes without ER, we next evaluated protein phosphorylation post exposure to FSS in the triple negative breast cancer (TNBC) cell line MDA-MB-231. Results demonstrated elevated p-mTOR (Ser2448) at 1 and 2 weeks post exposure to FSS; however, the factor increase was variable for each replicate and not significant (Figure 4d). The TNBC cell line demonstrated no change in phosphorylation of p-AKT (Ser473), p-AKT1S1 (Thr246), or p-ERK1/2 (Thr202/Tyr204).

**Figure 5:**
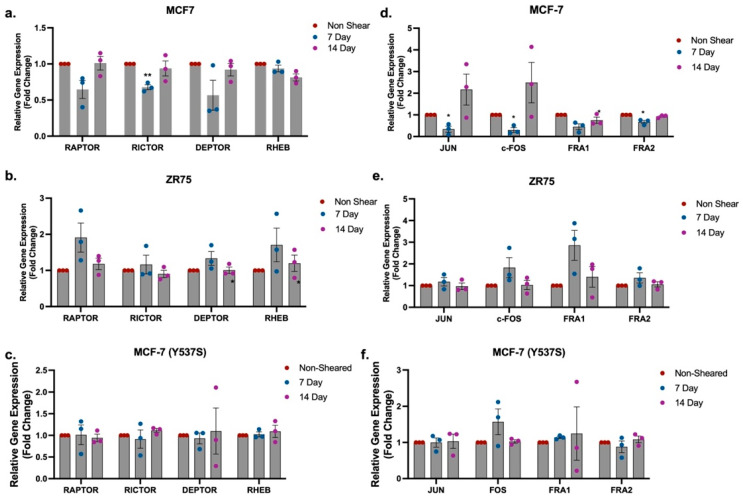
Exposure to fluid shear stress does not alter transcription of mTOR- and MAPK-associated genes in hormone receptor positive breast cancer. (**a**–**f**) Gene expression in HR positive breast cancer cell lines MCF-7 (**a**,**d**), ZR-75 (**b**,**e**), and MCF-7-Y537S (**c**,**f**). Normalization was to non-sheared MCF-7 cells maintained in suspension followed by seeding in culture on TCP for 7 and 14 days. While in culture, cells were not exposed to FSS. Error bars represent SEM and * *p* < 0.05 and ** *p* < 0.01.

Taken together, the phospho-protein Western blot data demonstrates an increase in p-mTOR but no change in p-AKT (Ser473) or p-ERK (Thr202/Tyr204) signaling. While our prior studies demonstrated an increase in p-AKT (Ser473) and repression in p-ERK (Thr202/Tyr204) immediately after exposure to FSS, this was not observed following culture of cells for 1 week and beyond. We next evaluated gene expression for genes associated with both the mTOR and ERK1/2 signaling pathways following growth on TCP for 1, 2, and 3 weeks post exposure to FSS. Results demonstrated that the mTORC2-associated gene *RICTOR* was significantly repressed by a factor of 0.68 ± 0.03 in MCF-7 cells following 1 week of growth after exposure to FSS compared to non-FSS-exposed control cells (Figure 5a). This trend was not observed in the ZR-75 or MCF-7-Y537S cell lines (Figure 5b,c). Exposure to FSS significantly repressed the MAPK effectors *JUN*, *c-FOS*, and *FRA2* by factors of 0.34 ± 0.14, 0.30 ± 0.11, and 0.67 ± 0.07, respectively, in MCF-7 cells at 1 week post exposure to FSS compared to non-FSS-exposed control cells (Figure 5d). There were no changes to MAPK effectors in the ZR-75 or MCF-7-Y537S cell lines (Figure 5e,f).

## 3. Discussion

Metastatic HR^+^ breast cancer has a response rate of 30% to endocrine therapy [29], suggesting the need to better understand signaling cascades activated in HR^+^ breast cancer in the metastatic setting. To better inform signaling cascades activated during cancer metastasis, we performed proteomics on HR^+^ breast cancer cells exposed to FSS. Our proteomics data demonstrated enhanced phosphorylation of proteins associated with growth factor signaling cascades with observed increases in phosphorylated HER2, AKT1S1, RET, and RPTOR (Figure 1). Further, Western blot confirmed the activation of p-mTOR in HR^+^ breast cancer cell lines with WT ER but not in the mutant ER cell line. The increased phosphorylation of mTOR signaling is in accordance with prior studies that demonstrated metastatic HR^+^ breast cancer had amplifications and mutations to the AKT pathways [29]. Further, activation of the AKT/mTOR pathway is observed in metastatic tumors [30,31,32], and increased phosphorylation of AKT, mTOR, and HER2 correlate with poor outcome for disease-free survival [33]. While additional tests are required, the data presented here would suggest exposure to FSS-induced mTOR signaling in the metastatic setting in HR^+^ cancer cells with WT ER. Evaluation of prior published work on the MCF-7-Y537S mutant vs. WT MCF-7 cells demonstrates that the ER mutant cell line has elevated pathways associated with mTORC1 signaling [4,34]. Further, our phospho-proteomics studies demonstrated elevated levels of proteins in pathways associated with estrogen response and the p53 pathway. These pathways are also observed to be different in the MCF-7-Y537S and MCF-7 WT ER cell lines. While additional studies are required, the study performed draws initial insight on FSS survival pathways that may be enhanced in ER mutant cell lines.

**Figure 6:**
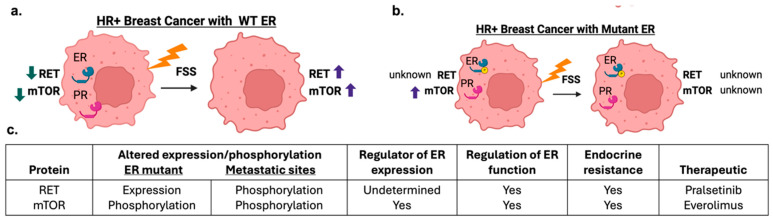
Overview of FSS impact on HR^+^ breast cancer. (**a**,**b**) Proposed pathway activity and HR expression in WT ER (**a**) and mutant ER (**b**) breast cancer cell lines before and after exposure to FSS. Made with Biorender. (**b**) Proposed protein targets of FSS and the known association with HR expression, metastatic expression, and potential therapeutic targets. (**c**) Known associations of ER with RET and mTOR in primary and metastatic HR^+^ breast cancer.

In addition to mTOR, the increased phosphorylation of HER2 signaling is also in accordance with prior studies, where metastatic HR^+^ breast cancer had enhanced HER2 signaling [29]. The observed increased p-HER2 (S807) in the proteomics data is not currently well described or documented in tumor data. The evaluation of HER2 phosphorylation in HER2 low tumors warrants further investigation to enhance targeted therapy options for metastatic setting in HR^+^ breast cancer. Prior clinical trials evaluating treatment of breast cancer in the metastatic setting demonstrated that breast cancer patients with low HER2 expression responded well to trastuzumab deruxtecan with increased overall patient survival [35]. While not evaluated in this study, additional phospho-protein targets of interest from the proteomic data included p-RET (S699). Prior RNA sequencing studies of primary and matched brain metastasis have identified RET as a highly upregulated kinase in breast cancer [27]. Further, RET expression is observed to correlate with ER expression and activate ER phosphorylation [36]. A direct link between RET and HR expression has not yet been made; however, RET is observed to be elevated in ER^+^ luminal B breast cancers, which historically have loss of PR [27]. The link between RET and ER^+^ breast cancer suggests RET expression can modulate breast cancer cell motility and metastasis [36,37]. Further studies are needed to evaluate the connection between RET phosphorylation and patient outcome [38]. While not yet in clinical trial for breast cancer, RET has recently been propsed as a novel target for ER fusion and mutant breast cancers [27,39]. RET activity was not further evaluated in this study; however, future studies should aim to determine the role of RET and HR expression (Figure 6). In addition to defining phospho-proteins altered with exposure to FSS, this study demonstrates loss of HR expression in WT ER cell lines following exposure to FSS (Figure 3). Further, the ER mutant cell line that displays constitutive active ER did not demonstrate a loss of ER or PR expression. Loss of both PR and ER is more commonly observed in metastatic tumors compared to matched primary tumors. In a meta-analysis of 39 studies in the metastatic setting, 22.5% of tumors converted from ER^+^ to ER^−^ between the primary and secondary site [40]. Others found that 30.63% of ER^+^ and 33.97% of PR^+^ patients’ primary tumors converted from positive to negative after metastasis [41]. The positive to negative switch of both ER and PR were also associated with worse survival when compared to persistent positivity [41]. The conversion of HR^+^ primary breast tumors to HR^−^ in the secondary site has been documented by many clinical studies [40,41]. To date, it is undetermined what causes this cellular loss of HR. One mechanism may be in the increased phosphorylation of p-mTOR in HR^+^ breast cancer cells following exposure to FSS. HER2 and mTOR are known mediators of PR expression and these signaling pathways regulate ER function [42,43]. The initial results of this study suggest FSS activation of growth factor signaling and loss of HR expression; however, one limitation to this study was the collection and evaluation of cells through bulk analysis. Our prior work utilizing single cell evaluation of cancer cells exposed to FSS demonstrated that the levels of p-AKT and p-mTOR activation varies at the single cell level [22]. Additional studies interrogating protein activation and HR expression on select cell populations would enhance the understanding of the impact of FSS on HR expression in the metastatic setting. Further, the observed variability from the bulk analysis and loss of protein activation over time may be dampened through additional single cell analysis. The study presented here provides initial groundwork for uncovering mechanisms of hormone receptor conversion and suggests that exposure to FSS induces the activation of growth factor signaling; specifically, the data suggest AKT/mTORC1 signaling is activated by FSS. Currently, both mTOR inhibitors and inhibitors to upstream mTOR mediators, such as RET, are candidate therapies for metastatic HR^+^ breast cancer [27,39] (Figure 6).

## 4. Materials and Methods

### 4.1. Cells and Culture Reagents

MCF7, ZR75, and MDA-MB-231 human breast cancer cells were purchased from ATCC. The MCF-7-Y537s human breast cancer cells were provided by the lab of Dr. Simak Ali. MCF-7-Y537S cells were generated and maintained as previously described [26]. All other cell lines were cultured in Dulbecco’s Modified Eagle Medium (DMEM; Gibco, Waltham, MA, USA, 11965-092) supplemented with 10% *v/v* HyClone Cosmic Calf Serum (Cytiva SH30087.03, Marlborough, MA, USA), 1% MEM Amino Acids (Gibco, 11130051), 1% MEM Non-Essential Amino Acids (Gibco, 11140076), 1% Antibiotic/Antimycotic (Gibco, 15240-062), and 0.048 μg/mL Insulin (Gibco, 12585-014). Cells were maintained in T-182.5 (VWR, Radnor, PA, USA) flasks in a humidified incubator at 37 °C and 5% *v/v* CO_2_. The cells were subcultured when confluent by first washing the cells with 1X phosphate-buffered saline (PBS: 137 mM NaCl, 10 mM Na_2_HPO_4_, 27 mM KCl, and 1.75 mM KH_2_PO_4_ at pH 7.4) and then detaching the cells with 3.7 mM UltraPure™ 0.5 M EDTA, pH 8.0 (Thermofisher, Waltham, MA, USA 15575020) diluted in 1X PBS. Cells were then transferred into a new cell culture flask. Cells used for experimentation were from flasks at 80–90% confluence. They were lifted by first washing cells with 1X PBS and then detaching them with StemPro Accutase (Thermofisher, A1110501). For the estrogen stimulation experiments, HR^+^ cancer cells were placed in media free of exogenous estrogens, phenol free, and supplemented with 5% Dextran stripped FBs, 1% glutamax, 1% MEM Amino Acids, and 1% MEM Non-Essential Amino Acids (Gibco, Billings, MT, USA 11140076) 24 h prior to the shearing event. Those cells were sheared and plated in stripped media until collection. Cells sheared for 1-, 2- and 3-week timepoints were plated in full estrogenic media in a T182.5 flask until collection.

### 4.2. Exposing Cells to Fluid Shear Stress Using the Microfluidic Device

The design and fabrication of the silicon master wafer used for the devices is described in our prior work [22]. The shearing microfluidic device consisted of a single fluidic channel (1 m long, 70 µm wide, and 100 µm tall) fabricated by polydimethylsiloxane (PDMS) replication from the silicon wafer. The PDMS replicas (Sylgard 184, Ellsworth Adhesives, Germantown, WI, USA) were made by mixing a 10:1 ratio of base to curing agent followed by degassing in a vacuum chamber to delete any bubbles. The PDMS mixture was poured into the silicon master and cured for 12 h at 65 °C. Once completed, the PDMS replicas were cut to size with an X-Acto knife and removed from the silicon master. The inlet and the outlet were made by using a blunted 18-gauge needle. Finally, the PDMS replica was bonded to a 25 mm × 75 mm glass slide using an O_2_ Harrick Plasma PDC-32G basic plasma cleaner (Harrick Plasma, Ithaca, NY, USA) for 2 min and 30 s and then exposed to plasma for 15 s. the devices were left for at least 24 h to ensure proper bonding between PDMS and glass. A 15-cm-long section of Tygon tubing (0.022” inner diameter × 0.042” outside diameter, Cole-Parmer, Vernon Hills, Illinois, USA) per device was cut and used to connect the inlet of the device to a 23-gauge needle connected to a 1 mL syringe. A length of 14 cm of tubing was used to connect the outlet of the device to a microcentrifuge tube to collect the cells post shearing. To prevent clumping and help maintain a single-cell suspension inside the syringe, all cell suspensions were diluted to 500,000 cells per 1 mL syringe and supplemented with 0.5% Pluronic™ F-68 Non-ionic Surfactant (100X) (Thermofisher, #24040032). A 10-syringe syringe pump (KDS 220CE, KD Scientific, Holliston, MA, USA) was used for all FSS exposure experiments to allow for an increased number of shearing devices per experiment to obtain sufficient cellular yields for all proteomics, gene expression, and Western blot studies.

### 4.3. Proteomics

Proteomics was performed through core services provided by the IDeA National Resource for Quantitative Proteomics. The samples used for proteomic/phospho-proteomic analysis were MCF-7 cells stripped for 24 h prior to the shearing event. The cells were loaded into the 1 mL syringe at a concentration of 500,000 cells/mL of stripped media, sheared, and collected immediately (on ice) post-shear.

In brief, methods as provided by the core are: CME bHPLC phosphoTMT Methods—Orbitrap Eclipse was performed through the following methods. Total protein from each sample was reduced, alkylated, and purified by chloroform/methanol extraction prior to digestion with sequencing grade modified trypsin/LysC (Promega, Madison, WI, USA). Tryptic peptides were labeled using a tandem mass tag 10-plex isobaric label reagent set (Thermo) and enriched using High-Select TiO_2_ and Fe-NTA phospho-peptide enrichment kits (Thermo) following the manufacturer’s instructions. Both enriched and un-enriched labeled peptides were separated into 46 fractions on a 100 × 1.0 mm Acquity BEH C18 column (Waters, Milford, MA, USA) using an UltiMate 3000 UHPLC system (Thermo) with a 50 min gradient from 99:1 to 60:40 buffer A:B ratio under basic pH conditions, and then consolidated into 18 super-fractions. Each super-fraction was then further separated by reverse phase XSelect CSH C18 2.5 um resin (Waters) on an in-line 150 × 0.075 mm column using an UltiMate 3000 RSLCnano system (Thermo). Peptides were eluted using a 75 min gradient from 97:3 to 60:40 buffer A:B ratio. Eluted peptides were ionized by electrospray (2.4 kV) followed by mass spectrometric analysis on an Orbitrap Eclipse Tribrid mass spectrometer (Thermo) using multi-notch MS3 parameters. MS data were acquired using the FTMS analyzer in top-speed profile mode at a resolution of 120,000 over a range of 375 to 1500 *m*/*z*. Following CID activation with normalized collision energy of 31.0, MS/MS data were acquired using the ion trap analyzer in centroid mode and normal mass range. Using synchronous precursor selection, up to 10 MS/MS precursors were selected for HCD activation with normalized collision energy of 55.0, followed by acquisition of MS3 reporter ion data using the FTMS analyzer in profile mode at a resolution of 50,000 over a range of 100–500 *m*/*z*.

- Buffer A = 0.1% formic acid, 0.5% acetonitrile
- Buffer B = 0.1% formic acid, 99.9% acetonitrile
- Both buffers adjusted to pH 10 with ammonium hydroxide for offline separation
- Data analysis—phosphoTMT

Proteins were identified and reporter ions quantified by searching the UniprotKB database restricted to Homo sapiens (June 2021) using MaxQuant (Max Planck Institute, version 2.0.3.0) with a parent ion tolerance of 3 ppm, a fragment ion tolerance of 0.5 Da, a reporter ion tolerance of 0.001 Da, trypsin/P enzyme with 2 missed cleavages, variable modifications including oxidation on M, Acetyl on Protein N-term, and phosphorylation on STY, and fixed modification of Carbamidomethyl on C. Protein identifications were accepted if they could be established with less than 1.0% false discovery. Proteins identified only by modified peptides were removed. Protein probabilities were assigned by the Protein Prophet algorithm [44]. TMT MS3 reporter ion intensity values are analyzed for changes in total protein using the unenriched lysate sample. Phospho (STY) modifications were identified using the samples enriched for phosphorylated peptides. The enriched and un-enriched samples are multiplexed using two TMT10-plex batches, one for the enriched and one for the un-enriched samples.

Following data acquisition and database search, the MS3 reporter ion intensities were normalized using ProteiNorm [45]. The data were normalized using cyclic loess [46] and analyzed using proteoDA to perform statistical analysis using Linear Models for Microarray Data (limma) with empirical Bayes (eBayes) smoothing to the standard errors [46,47]. A similar approach is used for differential analysis of the phospho-peptides, with the addition of a few steps. The phospho-sites were filtered to retain only peptides with a localization probability of >75%, filter peptides with zero values, and log2 transformed. Limma was also used for differential analysis. Proteins and phospho-peptides with an FDR-adjusted *p*-value < 0.05 and an absolute factor change >2 were considered significant.

### 4.4. Western Blotting

A 10-syringe syringe pump (KDS 220CE, KD Scientific) was used for FSS exposure, providing shearing events of multiple microfluidic devices at one time. After passing through the shearing device (sheared) or suspended (non-sheared), cells were seeded on tissue culture plastic and grown in culture. Cells exposed to FSS were pooled from multiple microfluidic devices to achieve appropriate cell numbers for growth in culture. Cells were lysed using 150 µL Mammalian Protein Extraction Reagent (M-PER) (Thermofisher 78501) with 1X protease (Thermofisher 1862209) and phosphatase inhibitors (Thermofisher 1862495). The lysed pellets were then centrifuged at 10,000 rpm at 4 °C for 10 min. A standardized amount of total protein was added to a new 1.5 mL microcentrifuge tube, ~20 µg per well. Reducing agent (Life Technologies, Carlsbad, CA, USA, B0009) and NuPAGE LDS sample buffer (Life Technologies B0007) were added to the samples per manufacturer’s protocol then the proteins were heat denatured at 100 °C for 10 min. The samples were then run on Bis-Tris-NuPAGE gel (Invitrogen, Grand Island, NY, USA) in the Invitrogen mini gel tank (A25977) at 100 V for 1 h. Using iBlot and iBlot transfer stacks per manufacturer’s protocol (Invitrogen, Grand Island, NY, USA), protein was transferred to nitrocellulose from the gels. The blots were blocked by incubation in 3% milk. After blocking, the membrane was incubated with primary antibody overnight at room temperature for p-AKT (Ser473) (Cell Signaling Technologies, Danvers, MA, USA, #4060), p-pras40(Thr246) (Cell Signaling Technologies, #2997), p-mTOR (Ser2448) (Cell Signaling Technologies, #5536), and p-ERK1/2 (Thr202/Tyr204) (Cell Signaling Technologies, #9101) (diluted 1:1000 in 3% milk). After incubation with primary antibody, the membrane was washed in 1X TBS-T three times for ten minutes each and incubated for 1 h in IRDye^®^ 800 CW secondary antibody (LI-COR Bioscience, Lincoln, NE, USA) at room temperature (1:10,000 dilution in 3% milk). Next, the nitrocellulose blotting papers were washed three times for ten minutes each in 1X TBS-T. Band density was determined using a LI-COR Odyssey imager. Rho GDI-α (Santa Cruz Biotechnology, Santa Cruz, CA, USA sc-373724) diluted 1:500 in 3% milk was utilized as the protein loading control. Normalization was to loading control (Rho GDI-α).

### 4.5. qRT-PCR

The 10-syringe syringe pump described above was used for FSS exposure. After passing through the shearing device (sheared) or suspended (non-sheared), cells were either (1) collected immediately after FSS exposure on ice in a 1.5 mL microcentrifuge tube or (2) seeded on tissue culture plastic and grown in culture. Cells exposed to FSS were pooled from multiple microfluidic devices to achieve appropriate cell numbers for growth in culture and RNA. For estrogen-mediated gene expression, 24 h prior to the shearing event, the cells were washed with 1X PBS and stripped media was applied to the surface of the growth flask (TCP). The cells were then sheared in stripped media and directly plated in T25 cell culture flasks in stripped media and allowed to adhere for 24 h. Cells were then treated with 100 µM 4-hydroxytamoxifen or Fulvestrant (Selleckchem, Houston, TX, USA), and after four hours of treatment, cells were treated with 100 pM 17β-estradiol (E2) (Sigma-Aldrich St. Louis, MO, USA) or vehicle control. After 24 h, cells were collected for RNA extraction with a Qiagen RNeasy kit (Qiagen, Valencia, CA, USA) per manufacturer’s protocol, cDNA synthesis was performed with a BioRad iscript cDNA supermix (BioRad, Hercules CA, USA) from 1000 μg total RNA, and qRT-PCR was performed for relative gene expression of ER, PR, and SDF1 with a BioRad CXC96 thermocycler run using the manufacturer’s protocol and BioRad Sybr green (BioRad, Hercules, CA, USA). For non-estrogen-mediated gene expression studies, cell pellets were collected in 10% FBS, and RNA extraction and cDNA synthesis was performed as described above. Genes analyzed were *ER* (F-GGCATGGTGGAGATCTTCGA, R-CCTCTCCCTGCAGATTCATCA), *PR* (F-TACCCGCCCTATCTCAACTACC, R-TGCTTCATCCCCACAGATTAAACA), *SDF1* (F-ACACTCCAAACTGTGCCCTTCA, R-CCACGTCTTTGCCCTTTCATC), *GPR30* (F-CTGCTTCTGTTTCGCGGATG, R-ACCCGGACAATGAGGGAGTA), *JUN* (F-GAGCTGGAGCGCCTGATAAT, R-CCCTCCTGCTCATCTGCAC), *c-FOS* (F-CAGACTACGAGGCGTCATCC, R-TCTGCGGGTGAGTGGTAGTA), *FRA1* (F-CGAAGGCCTTGTGAACAGAT, R-CTGCAGCCCAGATTTCTCAT), *FRA2* (F-CATCTCCCTCCGAATCCTGC, R-AGTGGGGGAGTTCAAGGAGT), *RPTOR* (R-, F-), *RICTOR* (F-TTCGTCTTCCTCTACCTGTTGTG, R-TTGGCAAGCAGTGTAAATGATGG), *DEPTOR* (F-CACCATGTGTGTGATGAGCA, R-TGAAGGTGCGCTCATACTTG), and *RHEB* (F-ATCCTCTCCTTCCACCCTCAAATC, R-AGCCACCAAACAAATGAAATCCC). Normalization was to *β-ACTIN* (F-TGAGCGCGGCTACAGCT, R-CCTTAATGTCACACACGATT) housekeeping gene and non-sheared control cells designated as 1. Factor change was determined using the 2-ΔΔCt method. Significant difference was *p*-value < 0.05.

### 4.6. Crystal Violet Cell Stain

After shearing at a density of 500,000 cells/mL, 10,000 cells per well were plated in triplicate in a 96-well plate in stripped media. They were drugged at a final concentration of (100 pM) 17β-estradiol, (100 nM) Tamoxifen, and (100 nM) Fulvestrant (ICI) in the 96-well plate after 24 h in culture. The inhibitors were added 4 h before the E2, and DMSO was used as a vehicle control. Cells were collected after 3 days in culture and the plates were stained using 0.33% crystal violet in 20% methanol. The stains were quantified by eluting in 1% SDS and reading at 570 nm on a 96-well plate spectrophotometer.

### 4.7. Statistical Analysis

One sample t and Wilcoxon tests were used to analyze the statistical significance between non-sheared (suspended) populations of cells and sheared populations (Graph Pad Prism V.4). *p*-values < 0.05 were considered statistically significant.

## Data Availability

Proteomic data is deposited in MassIVE with data set identifiers: MassIVE MSV000092544 PRIDE PXD044154 (ftp://massive.ucsd.edu/MSV000092544/) (accessed on 10 April 2024).

## Supplementary Materials

The following supporting information can be downloaded at: https://www.mdpi.com/article/10.3390/ijms25137119/s1.
