# Supplementary material for: Loss of Hormone Receptor Expression after Exposure to Fluid Shear Stress in Breast Cancer Cell Lines
Source: Int J Mol Sci. 2024 Jun 28;25(13):7119. doi: 10.3390/ijms25137119 (PMC11240898; doi:10.3390/ijms25137119)
Supplement: Supplementary file 1 [file ijms-25-07119-s001.zip › ijms-2946619-supplementary.pdf]

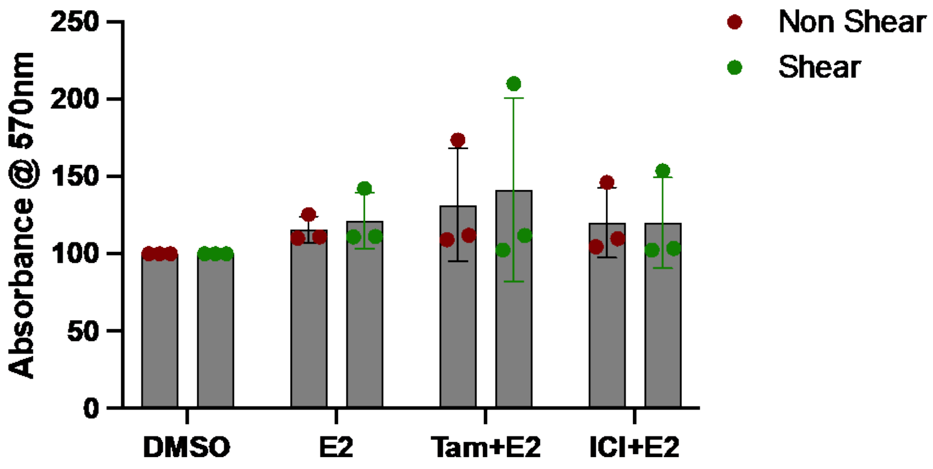

Supplemental Figure S1. FSS does not alter HR+ proliferation immediately after FSS exposure. MCF-7 cells were grown in media free of exogenous estrogens for 24 hours and then exposed to FSS followed seeding on TCP for 24 hours and then treatment with vehicle control (DMSO), 17-βestradiol (E2), or pre-treatment with tamoxifen or fulvestrant (ICI) prior to stimulation with E2. Cells were allowed 3 days of grow and then collected at end point for crystal violet stain. Error bars represent SEM. Non-Shear cells were MCF-7 cells maintained in suspension during the time as FSS exposure.
